# ASO Author Reflections: Genetic Counseling, a Path to Testing

**DOI:** 10.1245/s10434-024-15561-w

**Published:** 2024-06-05

**Authors:** Julie B. Siegel, Kevin S. Hughes, Andrea M. Abbott

**Affiliations:** Department of Surgery, Medical University of South Carolina, Charleston, SC USA

## Past

The accessibility of genetic testing for breast cancer patients has dramatically changed over the past decade. In 2013, the Supreme Court ruled that genes were not patent eligible, which led to an open market for the development of commercialized genetic testing. In addition, the advent of next-generation sequencing has allowed for multigene panels and more economical testing.^1^ High penetrance genes for breast cancer increase the likelihood of developing breast cancer by 5–20 times, and increased surveillance and management recommendations for these patients may have a significant impact.^2^ Therefore, it is imperative that high-risk patients undergo genetic testing. Despite growing public awareness, increased availability of testing, and reduction in cost, disparities persist in rates of genetic testing.^3^

## Present

To further understand what barriers exist that continue to impede uptake of genetic testing, we evaluated the rates of genetic counseling attendance and genetic testing for patients with a personal or family history of breast cancer. With an overall cohort attendance rate of nearly 50%, we found that black patients were significantly less likely to attend their genetic counseling appointment compared to white patients. Having a positive family history of breast cancer was a significant predictive factor for attendance regardless of race. Patients who were referred because of a significant family history (without a personal history of breast cancer) or a benign breast diagnosis were less likely to attend their counseling appointments. No socioeconomic factors were found to impact attendance rate. Once patients attended their genetic counseling appointment, 84% (n = 248) had genetic testing done. There were no significant factors associated with receipt of genetic testing in multivariate analysis.^4^

## Future

Even with recent increases in genetic testing accessibility, we found that there continue to be racial disparities in genetic counseling attendance. However, disparities in genetic testing are not present once patients attend a genetic counseling appointment. This disappearance of racial disparities may be secondary to the education that is provided during the counseling appointment as the information provided may mitigate barriers, such as lack of knowledge about genetic testing and fear of discrimination. The barrier to the actual counseling appointment may be removed with point-of-care testing. Previous research has shown that point-of-care testing significantly improves rates of genetic testing,^5^ as it removes the inconvenience of a second clinic visit. Future research studying point-of-care genetic testing and utilizing open-ended questionnaires may help to determine why some patients decline testing when known barriers are eliminated. The use of artificial intelligence in areas with a paucity of genetic counselors and medical providers may also off-set access barriers. We hope the results in the current study help institutions to expand point-of-care testing to mitigate racial disparities and bolster genetic testing rates for all patients.
